# Health-related quality of life and its determinants among patients with diabetes mellitus in a selected hospital in Sri Lanka: a cross-sectional study

**DOI:** 10.1186/s12902-026-02476-8

**Published:** 2026-08-10

**Authors:** Tharuni Navoda Abeyratne, Damsi Dileka Jayakody, Thamalshee Perera, Shiran Lakkana Rathnayaka, Lahiru Sandaruwan Galgamuwa

**Affiliations:** 1 Department of Community Medicine, Faculty of Medicine, Sabaragamuwa University of Sri Lanka, Rathnapura, Sri Lanka; 2 Department of Parasitology, Faculty of Medicine, Sabaragamuwa University of Sri Lanka, Rathnapura, Sri Lanka

**Keywords:** Quality of life, Diabetes mellitus, Socio-demographic factors, Sri Lanka

## Abstract

**Introduction:**

Health-related Quality of Life (HRQoL) is an excellent measure of life expectancy, particularly for those with a specific condition. Evaluation of QoL facilitates clinical decision-making, enhances communication with the patient, and aids in predicting therapy response. QoL can forecast a person’s ability to control their illness and preserve their long-term health and well-being. This study aimed to determine HRQoL and its socio-demographic and clinical determinants among Diabetes Mellitus (DM) patients attending endocrinology clinics at a tertiary care hospital in Sri Lanka.

**Methodology:**

This study was conducted from March to April 2024. QoL was assessed using the WHOQOL- BREF questionnaire. Socio-demographic factors, clinical characteristics, and treatment options of DM patients were assessed in relation to the QoL. T-test and ANOVA used to determine the differences in the QOL domain. The Pearson correlation coefficient was used to determine the linear relationship between two QOL domains. Multiple Linear Regression was applied to examine the relationship between QOL domains and socio-demographic factors, clinical characteristics, and treatment options.

**Results:**

A total of 170 patients with DM (Female − 62%, age range – 18–87 years, mean age 60.9 (± 11.4) years) participated. The environment domain showed the highest mean of QOL (57.89 ± 15.02), followed by psychological health (57.44 ± 18.72), physical health (53.38 ± 20.67), and social relationship domain (52.12 ± 18.71). Age, education level, comorbidities, and complications were significantly associated with QOL in physical health, while age, education level, smoking, comorbidities, and complications were significantly associated with QOL in psychological health. Additionally, education level was significantly associated with social relationships and residential area, and education level and comorbidities were significantly associated with the environment domain.

**Conclusions:**

Findings emphasize the importance of addressing both medical and socio-environmental aspects in diabetes management to improve overall well-being. Understanding these variations can guide interventions aimed at enhancing the physical, psychological, social, and environmental domains of individuals living with DM.

**Clinical trial number:**

Not applicable.

**Supplementary Information:**

The online version contains supplementary material available at https://doi.org/10.1186/s12902-026-02476-8.

## Introduction

Diabetes Mellitus (DM) is rapidly emerging as a major global public health concern among adults aged 20 to 70 years. It shortens the life expectancy and contributes to serious, costly, and often life-threatening complications [1]. In 2021, the global prevalence of diabetes among individuals aged 20 to 79 was estimated at 10.5%, affecting approximately 536.6 million people [2]. This number is projected to rise to 643 million by 2030 and to 783 million by 2045 [2]. By 2025, over 75% of the global diabetic population is expected to reside in developing countries [3].

The incidence of DM in Sri Lanka has increased significantly in recent years, becoming a major public health concern, with its prevalence steadily rising due to urbanization, increasingly sedentary lifestyles, and shifts in dietary habits. In 2019, the prevalence of diabetes among adults aged 18 years and over in Sri Lanka was reported at 23.0% [4]. The prevalence increased with age, peaking at 43% among individuals aged 60–69 years. Uncontrolled diabetes is linked to a range of long-term complications, including cardiovascular disease, kidney failure, blindness, and limb amputations, all of which significantly diminish a person’s quality of life [5].

DM profoundly affects quality of life, imposing a daily burden of disease management and leading to numerous health complications, mental health challenges, social restrictions, financial pressures, and an increased risk of hypoglycemic emergencies [6]. Empirical evidence consistently highlights associations between Health-related quality of life (HRQoL) and factors such as retirement status, socioeconomic disadvantage, lower educational attainment, physical inactivity, gender, age, marital status, occupation, and income level [7–9]. Furthermore, disease-specific characteristics, including the presence of comorbidities, diabetes-related complications, treatment regimens, body mass index (BMI), HbA1c levels, and disease duration, have been shown to significantly impact HRQoL [10–14].

Comprehensive evaluation of HRQoL and its determinants is crucial for optimizing the clinical management of DM, as early identification of individuals at risk for diminished quality of life facilitates the implementation of targeted and effective interventions [15]. Despite the global significance of this issue, research examining the predictors of HRQoL among DM patients in Sri Lanka remains limited. Therefore, the present study aims to assess HRQoL and its key determinants among individuals attending diabetes follow-up clinics at a tertiary care hospital in Sri Lanka.

## Materials and methods

### Study design and population

A cross-sectional study was conducted to assess the quality of life (QOL) among patients living with type 2 diabetes mellitus (T2DM). The study was carried out at the Teaching Hospital Ratnapura between March and April 2024.

Patients were eligible for inclusion if they were 18 years of age or older, had been diagnosed with T2DM for at least ten years before the study, and were attending the hospital during the study period. Exclusion criteria included newly diagnosed patients, individuals living with diabetes for less than ten years, those who were severely ill, pregnant, under 18 years old, or unable to provide reliable responses to the questionnaire. We included patients who visited DM clinics during the study period and were invited to participate in the study. A total of 170 patients gave their consent to participate in the study. Therefore, a total of 170 patients with T2DM who visited the diabetes mellitus clinic in the study period were enrolled in the study.

### Measurement tools

The WHOQOL-BREF, a shortened version of the WHOQOL-100 developed by the World Health Organization (WHO), was utilized to assess participants’ Health-related quality of life (QOL) [16]. The WHOQOL is an instrument formed by the World Health Organization (WHO) that measures a variety of subjective characteristics of quality of life. The WHOQOL BREF covers four different domains of quality of life, namely: physical health, psychological well-being, social relationships, and environmental health [17]. Higher values of the total scores indicate a higher level of QoL. The scale has been validated in Sri Lanka [18]. Initially, the 26 items were scored on a scale ranging from 4 to 20, but these scores were subsequently transformed to a 0–100 scale to align with studies using the original WHOQOL-100. The Questionnaire was translated into both Tamil and Sinhala, which are the native languages of participants to increase understanding of the purpose of the questions.

Following approval for data collection, all DM patients attending the clinic who met the inclusion criteria were invited to take part in the study. Informed consent was obtained from each participant prior to conducting interviews. For individuals who were unable to read the consent forms, the researcher provided a detailed verbal explanation to ensure full understanding before obtaining their signature. The questionnaires were administered and completed by the researchers through face-to-face interviews. Then, all completed questionnaires were approved by the participants after giving enough time to observe their answers.

After obtaining informed consent, participants were interviewed in hospital clinic settings. Data were collected through a structured questionnaire designed to gather information on quality of life, socio-demographic factors, and morbidity status. Collected details included age, gender, educational attainment, family structure, marital status, place of residence, family history of diabetes, duration since diagnosis, smoking habits, and exercise (30 min per day for 5 days per week).

Participants were classified as having diabetes-related complications if they reported experiencing at least one of the following conditions: nephropathy, retinopathy, neuropathy, hyperglycemia, coronary artery disease (CAD), hypoglycemia, or diabetic foot.

Diabetic kidney disease is a clinical diagnosis with diabetes, with albuminuria and/or reduced estimated glomerular filtration rate (eGFR), when an alternative renal pathology is clinically unlikely. Diabetic retinopathy is a common and highly specific neurovascular complication of diabetes. Chronic hyperglycaemia, which activates multiple pathways leading to abnormal vascular permeability, occlusion with ischemia, and subsequent neovascularization, drives the development and progression of DR. Diabetic neuropathy is defined as signs and symptoms of peripheral nerve dysfunction in people with diabetes in whom other causes of peripheral nerve dysfunction have been excluded. At the Endocrinology Clinic, diabetic patients with a history of more than 5 years are referred to the Ophthalmology Clinic and the Nephrology Clinic to identify possible microvascular complications. By reviewing their medical records, we were able to identify patients who had developed such complications.

### Statistical analysis

The data were initially entered into Microsoft Excel and subsequently imported into SPSS version 21 for statistical analysis. Descriptive statistics summarized the participants’ general characteristics. For continuous variables, the results were expressed as mean and standard deviation (SD), whereas categorical variables were described using frequencies and percentages. T-test and ANOVA are used to determine whether the differences in QOL domain between two groups or more than two groups respectively are statistically significant. After applying ANOVA, a post hoc analysis was performed using Tukey’s HSD (Honestly Significant Difference) test to identify which particular group means differed significantly from one another. The Pearson correlation coefficient was used to assess the linear relationship between two QOL domains. Multiple Linear Regression was used to examine the relationship between QOL domains and independent (predictor) variables. p-value less than 0.05 was considered significant.

## Ethical consideration

Ethical approval for the study was obtained from the Ethics Review Committee of the Faculty of Medicine at Sabaragamuwa University of Sri Lanka (ERC/FOM-SUSL/J 30-01-24). All research activities complied with the ethical principles outlined in the most recent version of the Declaration of Helsinki. Individuals diagnosed with diabetes mellitus who met the inclusion criteria and were available during the study period were invited to participate. Informed consent was secured from each participant prior to data collection. For those unable to read the consent forms, the researcher read and explained the information to ensure full understanding. Written consent was obtained only after confirming that participants had comprehended the details.

## Results

A total of 183 eligible patients were invited to the study. However, a total of 170 patients, all aged 18 years or older, participated in the study, with a response rate of 93%. The mean age of participants was 60.90 years (SD ± 11.42), and all had been diagnosed with diabetes for more than 10 years. The majority were female (62.4%), aged between 51 and 70 years (58.8%), living in rural areas (68.2%), married (94.1%), and unemployed (68.8%). Participants’ ages ranged from 18 to 87 years.

In terms of educational attainment, a total of 57.6% of participants had completed their education between Grade 6 and Grade 11. In addition, 64.7% of study participants indicated that one or more family members had diabetes. Only 15.3% had a history of smoking, while 17.6% reported engaging in physical activity for 30 min per day, five days a week.

Regarding the time duration of diabetes, 60.6% had been diagnosed between 10 and 14 years prior. The most common diabetes-related complications were retinopathy (56.5%) and neuropathy (54.7%), whereas cardiovascular complications were the least frequently reported (14.1%). Hypertension was the most prevalent comorbidity (70.6%), while liver disease was the least common (7.6%).

In terms of treatment, the majority of participants (66%) managed their diabetes with a combination of lifestyle modifications and oral antidiabetic medications. The fewest participants were using a combination of lifestyle modifications and insulin therapy (Table 1).

**Table 1:** Socio-demographic characteristics of study participants

Variables	Category	No. of participants (%)
Gender	Male	64 (37.6)
	Female	106 (62.4)
Age (Years)	≤ 50	30 (17.6)
	51–70	100 (58.8)
	≥ 70	40 (23.5)
Marital status	Married	160 (94.1)
	Unmarried	10 (5.9)
Occupation status	Employed	53 (31.2)
	Unemployed	117 (68.8)
Residence	Urban	54 (31.8)
	Rural	116 (68.2)
Education level	≤ Grade 5	30 (17.7)
	Grade 6–11	98 (57.6)
	≥ Advanced level	42 (24.7)
Family history of DM	Yes	110 (64.7)
	No	60 (35.3)
Smoking	Yes	26 (15.3)
	No	144 (84.7)
Exercise	Yes	30 (17.6)
	No	140 (82.4)
Duration of diagnosis (Years)	14-Oct	103 (60.6)
	15–19	28 (16.5)
	20–24	22 (12.9)
	≥ 25	17 (10)
Comorbidity	Hypertension	120 (70.6)
	CVD	38 (22.4)
	CKD	35 (20.6)
	Liver disease	13 (7.6)
	Others	72 (42.4)
Complication	Retinopathy	96 (56.5)
	Hyperglycemia	51 (30)
	Neuropathy	93 (54.7)
	Cardiovascular	24 (14.1)
	Diabetic foot	35(20.6)
	Nephropathy	40 (23.5)
	Hypoglycemia	44 (25.9)
Treatment type	Lifestyle Modifications +	113 (66.5)
	Oral Antidiabetic Medication	
	Lifestyle Modifications + Insulin	18 (10.6)
	Lifestyle Modifications +	39 (22.9)
	Oral Antidiabetic Medication + Insulin	

Table 2 shows the quality of life scores. Regarding the different quality of life domain scores, the environmental domain was found to have the highest score (57.9 ± 15.0), followed by psychological health (57.4 ± 18.7), physical health (53.4 ± 20.7), and social relationship domain (52.1 ± 18.7) (Table 2).

**Table 2:** Scores for QOL domains

Domain	Minimum	Maximum	Mean	SD
Physical Health	0	100	53.4	20.7
Psychological Health	0	100	57.4	18.7
Social Relationships	0	100	52.1	18.7
Environment	0	100	57.9	15

In the present study, patients of a young age had significantly higher QOL scores in physical health and psychological well-being compared to older patients (*p* = 0.035 and *p* = 0.048, respectively). Patients with higher educational attainment (advanced level or university education) reported significantly better QOL scores in physical health (*p* < 0.001), psychological health (*p* < 0.001), social relationships (*p* = 0.029), and the environment domain (*p* < 0.001) compared to those with lower education levels.

Urban residents had higher QOL scores in the psychological health and environmental domains than those living in rural areas (*p* = 0.023 and *p* < 0.001, respectively). Furthermore, there were statistically significant differences in the physical health, psychological health, and social relationship domain scores based on marital status (*p* = 0.024, *p* = 0.021, *p* = 0.034, respectively). Patients who exercised for at least 30 min per day, five days a week, reported significantly higher QOL scores in psychological health, social relationships, and the environmental domain compared to those who did not exercise daily (*p* = 0.04, *p* = 0.048, and *p* = 0.039, respectively). No statistically significant differences were found in QOL scores across any domain based on gender or occupation (Table 3).

**Table 3:** WHOQoL domains with socio-demographic characteristics (Mean (SD))

Variable	Category	Physical Health	Psychological Health	Social Relationships	Environmental
Age (Years)	≤ 50	58.0 (25.3)	61.3 (22.9)	52.9 (22.2)	55.6 (18.7)
	51-70	54.8 (19.3)	58.7 16.2)	53.6 (18.6)	59.113.3)
	≥ 70	43.3 (18.9)	51.320.1)	47.8 (15.6)	56.7 16.6)
	*p* value	0.035	0.048	0.24	0.462
Gender	Male	56.9 (19.1)	58.4(19.6)	49.6 (19.2)	58.4 (15.2)
	Female	51.3 (21.3)	56.9 (18.2)	53.7 18.3)	57.6 (14.9)
	*p* value	0.084	0.605	0.168	0.715
Marital status	Married	52.5 (20.3)	56.6 (18.5)	52.9 (18.6)	58.2 (14.7)
	Unmarried	67.7 (21.7)	70.7 (18.6)	55.2 (19.1)	53.3 (20.0)
	*p* value	0.024	0.021	0.034	0.32
Residential area	Urban	56.3 (20.7)	60.2 (17.2)	50.1 (19.5)	65.5 (13.6)
	Rural	52.0 (20.6)	55.2 (19.1)	53.1 (18.4)	54.3 (14.4)
	*p* value	0.214	0.023	0.348	< 0.001
Education Level	≤ Grade 5	42.5 (22.5)	45.7 (20.4)	47.3 (19.1)	45.1 (17.4)
	Grade 6-11	53.1 (19.6)	57.3 (17.5)	50.9 (18.0)	57.6 (11.6)
	≥ Advanced level	61.9 (18.4)	66.2 (15.7)	58.34 (18.9)	67.7 (13.3)
	*p* value	< 0.001	< 0.001	0.029	< 0.001
Occupation	Employed	56.3 (17.5)	60.4 (16.7)	55.7 (20.4)	56.8 (15.9)
	Unemployed	52.0 (21.9)	56.1 (19.5)	50.5 (17.7)	58.4 (14.7)
	*p* value	0.21	0.173	0.098	0.514
Family history of DM	Yes	51.6 (19.6)	56.8 (18.5)	53.3 (18.9)	58.4 (13.9)
	No	56.7 (22.3)	58.6 (19.3)	49.9 (18.4)	57.0 (17.0)
	*p* value	0.125	0.566	0.271	0.575
Smoking	Yes	54.0 (18.6)	52.8 (19.6)	54.0 (20.6)	54.3 (18.6)
	No	53.3 (21.1)	58.3 (18.5)	51.8 (18.4)	58.5 (18.3)
	*p* value	0.869	0.171	0.574	0.182
Exercise	Yes	59.5 (18.1)	63.8 (13.5)	58.2 (17.6)	63.0 (12.2)
	No	52.1 (21.0)	56.1 (19.4)	50.8 (18.8)	56.8 (15.4)
	*p* value	0.074	0.04	0.048	0.039

In post hoc Tukey HSD analysis, patients aged more than 70 years old had significantly less QOL than the other 2 groups (*p* < 0.05). Patients had education with Advanced level showed significantly higher QOL than the other two groups in physical, psychological, and environmental health domains (*p* < 0.05). When considering the social domain, patients with higher education was significant with patients who had education up to grade 5 (*p* = 0.035).

In relation to clinical features, a significant association was found between chronic kidney disease and both the physical and psychological health domains. Furthermore, dyslipidemia and cerebrovascular accidents were significantly associated with the physical, psychological, and environmental health domains.

When considering diabetes-related complications, patients with retinopathy exhibited significantly lower quality of life (QOL) scores in the physical and psychological health domains. Similarly, patients with neuropathy, cardiovascular disease, and nephropathy demonstrated significantly reduced QOL scores in the physical health domain. Additionally, a highly significant association was observed between diabetic foot complications and scores in the psychological health and social relationships domains. A strong statistical relationship was also found between psychological health and nephropathy complications (Table 4).

**Table 4:** WHOQoL domains with clinical characteristics (Mean ± SD)

Variable	Category	Physical Health	Psychological Health	Social Relationships	Environmental
Duration of Diagnosis (Years)	10–14	55.5 (21.2)	59.1 (18.6)	53.3 (18.0)	56.9 (15.0)
	15–19	55.5 (19.8)	57.1 (18.6)	49.8 (21.2)	60.2 (14.9)
	20–24	49.0 (20.3)	57.8 (18.5)	54.2 (17.7)	60.7 (17.4)
	≥ 25	42.8 (16.0)	47.1 (20.0)	45.9 (19.6)	56.6(12.2)
	*p* value	0.079	0.109	0.391	0.572
Comorbidities					
Hypertension	Present	52.1 (20.2)	55.7 (18.5)	52.9 (17.5)	58.1 (14.9)
	Absent	56.6 (21.5)	61.7 (18.8)	50.2 (21.4)	57.4 (15.4)
	*p* value	0.194	0.055	0.392	0.791
CVD	Present	50.4 (20.4)	56.1 (18.1)	49.4 (20.4)	56.3 (16.9)
	Absent	54.2 (20.8)	57.8 (19.0)	52.9(18.2)	58.314.5)
	*p* value	0.322	0.604	0.308	0.464
CKD	Present	41.7 (19.3)	46.5 (19.8)	51.6 (16.9)	56.0 (15.7)
	Absent	56.4 (20.0)	60.3 (17.4)	52.3 (19.2)	58.4 (14.9)
	*p* value	< 0.001	< 0.001	0.868	0.404
Liver disease	Present	51.7 (20.1)	54.9 (22.4)	44.3 (22.7)	56.9 (12.5)
	Absent	53.5 (20.8)	57.7 (18.5)	52.8 (18.3)	58.0 (15.2)
	*p* value	0.760	0.615	0.117	0.809
Others	Present	46.6 (19.8)	51.6 (18.3)	53.3 (18.4)	53.8 (15.3)
	Absent	58.4 (20.0)	61.7 (17.9)	51.3 (19.0)	60.9 (14.1)
	*p* value	< 0.001	< 0.001	0.505	0.002
Complications					
Retinopathy	Present	47.0 (19.0)	53.3 (18.6)	52.2 (16.8)	57.4 (13.7)
	Absent	61.7 (19.8)	62.8 (17.7)	52.0 (21.1)	58.6 (16.6)
	*p* value	< 0.001	0.001	0.937	0.602
Hyperglycaemia	Present	54.9 (18.1)	59.1 (16.1)	47.8 (18.8)	59.5 (12.4)
	Absent	52.7 (21.7)	56.8 (19.8)	54.0 (18.5)	57.2 (16.0)
	*p* value	0.532	0.464	0.046	0.378
Neuropathy	Present	49.3 (19.6)	55.6 (17.9)	52.2 (18.7)	59.5 (14.0)
	Absent	58.3 (21.0)	59.6 (19.6)	52.1 (18.8)	55.9 (16.1)
	*p* value	0.005	0.169	0.981	0.115
Cardiovascular	Present	45.4 (19.8)	53.0 (19.4)	48.5 (16.9)	59.7 (13.0)
	Absent	54.7 (20.6)	58.2 (18.6)	52.7 (19.0)	57.6 (15.3)
	*p* value	0.040	0.210	0.312	0.534
Diabetic foot	Present	48.0 (17.3)	49.9 (19.2)	43.7 (15.7)	56.2 (13.1)
	Absent	54.8 (21.3)	59.4 (18.2)	54.3 (18.9)	58.3 (15.5)
	*p* value	0.084	0.007	0.002	0.463
Nephropathy	Present	39.3 (16.2)	45.3 (18.3)	53.0 (16.4)	54.9 (14.2)
	Absent	57.7 (20.0)	61.2 (17.3)	51.9 (19.4)	58.8 (15.2)
	*p* value	< 0.001	< 0.001	0.730	0.150
Hypoglycaemia	Present	51.7 (20.0)	57.7 (18.9)	51.9 (17.3)	59.1 (14.0)
	Absent	54.0 (21.0)	57.4 (18.8)	52.2 (19.3)	57.5 (15.4)
	*p* value	0.539	0.923	0.913	0.541
Treatment type	1	52.9 (22.1)	56.7 (19.9)	51.9 (19.5)	56.6 (16.0)
	2	58.1 (17.3)	60.5 (16.8)	47.8 (16.2)	61.6 (12.9)
	3	52.5 (17.9)	58.1 (16.3)	54.7 (17.5)	59.9 (12.7)
	*p* value	0.598	0.711	0.435	0.277

Pearson correlation analysis was performed to examine the relationships between the QOL domains within the study group. The results indicated that physical health was significantly correlated with both psychological health and the environmental domain. Psychological health was also significantly associated with physical health, social relationships, and the environmental domain (Table 5).

The multiple linear regression analysis revealed that age, education level, comorbidities, and complications were significantly associated with QOL in the physical health domain. For the psychological health domain, significant factors included gender, education level, smoking status, comorbidities, and complications. Furthermore, education level was significantly associated with the social relationships domain, while residential area, education level, and comorbidities were significantly associated with the environmental domain (Table 6).

## Discussion

Measuring quality of life (QoL) in a population helps researchers and policymakers understand the broader effects of health and social policies. The WHOQoL tools help track changes in policies and measure quality of life in different groups of people and settings [19].

**Table 5:** Pearson correlation of study domains

Domain		Physical Health	Psychological Health	Social Relationships	Environment
Physical	Correlation coefficient	-	0.777	0.099	0.485
Health	p value	-	< 0.001	0.197	< 0.001
Psychological	Correlation coefficient	0.777	-	0.153	0.571
Health	p value	< 0.001	-	0.046	< 0.001
Social	Correlation coefficient	0.099	0.153	-	0.178
Relationships	p value	0.197	0.046	-	0.02
Environment	Correlation coefficient	0.485	0.571	0.178	-
	p value	< 0.001	< 0.001	0.02	-

The environmental domain had the highest average QoL score in the present study. It plays a crucial role in promoting a high quality of life (QoL) in diabetes patients by enhancing their physical, social, and emotional well-being. A supportive environment, such as living in a safe, accessible area with good healthcare services, can greatly improve diabetes management. Access to well-maintained public spaces, walking paths, and healthy food options makes it easier for diabetic patients to engage in regular physical activity and maintain a balanced diet [20]. Effective healthcare facilities, including nearby clinics and diabetes specialists, ensure timely interventions, helping patients maintain optimal health [21]. In this way, a positive environment enhances both the physical and psychological aspects of diabetes care, leading to a higher overall QoL.

**Table 6:** Multiple linear regression quality of life among study participants

Domain	Category	Unstandardized Coefficient (β)	95% CI (lower – upper limit)	p value
Physical	Age	-11.327	-18.176 - -4.479	0.001
health	Educational level	5.803	1.375 - 10.230	0.011
	comorbidity	-4.461	-6.407	0.007
	Complications	-4.787	-4.512	<0.001
Psychological	Age	-6.788	-12.482	0.033
health	Educational level	6.177	2.142 - 10.211	0.003
	Smoking	10.902	2.611 - 19.194	0.01
	comorbidity	-5.613	-5.839	<0.001
	Complications	-2.425	-4.111	0.021
Social	Marital status	-13.817	-23.807	0.023
relationship	Educational level	5.906	1.327 - 10.485	0.012
Environment	Residential area	-7.17	-8.765	0.002
	Educational level	9.226	5.984 - 12.468	<0.001
	comorbidity	-2.479	-4.692	0.038

In the present study, old age negatively affects QoL mainly due to worsening health, loss of independence, loneliness, financial stress, and mental health issues [22, 23]. As people age, they often face a greater burden of diabetes-related complications, such as vision problems, nerve damage, kidney disease, and cardiovascular issues, all of which can limit daily activities and independence. Management of DM becomes more challenging with age due to cognitive decline, which can affect medication adherence and blood sugar monitoring. Additionally, the psychological impact of living with a chronic illness, combined with social isolation, depression, or financial stress common in older age, can further lower emotional and mental well-being [24]. Together, these factors contribute to a decline in both physical and psychological aspects of QoL among elderly diabetic patients.

Higher education can have a positive effect on the quality of life (QoL) for DM patients in South Asian countries [25–27], which found that higher education is linked to better Health-Related Quality of Life (HRQoL). People with more education usually have a better understanding of their condition, which helps them manage their health more effectively. This includes regularly checking blood sugar, taking medications correctly, eating healthily, and exercising [28, 29]. Education also makes it easier to access health information and medical services, helping patients make better decisions and get medical help on time [30]. Additionally, higher education is often connected to financial stability, which can reduce the stress of paying for diabetes care. These factors combined lead to better physical health, stronger mental resilience, and better social relationships for people with higher education.

Living in an urban area can positively affect the quality of life (QoL) in patients with diabetes by providing better access to healthcare resources, education, and support services. Urban settings often have a higher concentration of specialized healthcare providers, diabetes clinics, and advanced medical facilities, allowing patients to receive timely and comprehensive care [22, 31]. In addition, urban areas typically offer more opportunities for diabetes education programs, support groups, and community activities, which can improve self-management skills and reduce feelings of isolation. These factors help diabetic patients living in urban environments maintain better control of their disease.

In the present study, Comorbidities have a major negative impact on the quality of life (QoL) in patients with diabetes. Many individuals with diabetes also suffer from other chronic conditions such as hypertension, cardiovascular disease, kidney failure, or depression. Managing multiple illnesses at once increases the complexity of treatment, raises the risk of medication side effects, and can lead to higher rates of hospitalization. Physical limitations from comorbidities often reduce mobility, energy levels, and the ability to perform daily activities, while mental health issues like anxiety and depression further strain emotional well-being [32, 33]. The combined burden of multiple health problems can overwhelm patients, making it harder to maintain good diabetes control and resulting in a significantly lower overall QoL.

Diabetes complications have negative impacts on the quality of life (QoL) of patients, affecting their physical health, emotional well-being, and social functioning. Lower scores on all HRQoL scales were linked to an increase in complications. These are inconsistent with previous studies [34–36]. Physical complications such as neuropathy, retinopathy, nephropathy, and cardiovascular disease often lead to chronic pain, loss of mobility, visual impairment, and fatigue, all of which can make daily tasks challenging or impossible without assistance. As complications worsen, the need for ongoing medical treatments and hospitalizations adds financial strain and increases emotional stress, further reducing overall QoL [37]. Furthermore, several studies reported that lower QOL scores were associated with having diabetes-related complications [38, 39].

Smoking negatively affects the psychological health of patients with diabetes. Nicotine and other chemicals in cigarettes can disrupt brain chemistry, exacerbating mood disorders and impairing cognitive function. The constant awareness of the health risks associated with smoking, combined with the physical limitations it causes, can create significant psychological distress [40]. Furthermore, smoking can hinder diabetes management by increasing insulin resistance, leading to poorer blood sugar control, which in turn heightens feelings of anxiety and concern about the future [41, 42].

Being unmarried can sometimes positively affect the social relationships and quality of life (QoL) in DM patients by allowing more independence and freedom to focus on personal health management. Without the complexities of a romantic relationship, individuals may find it easier to prioritize their self-care, including regular exercise, dietary choices, and adhering to their diabetes treatment plan [43]. Additionally, unmarried individuals may have the flexibility to build a broader and more diverse social network, including friends, family, and support groups, without the constraints or responsibilities that can come with a partner [44]. This wider social support can foster emotional well-being, reduce feelings of loneliness, and improve overall mental health, which in turn enhances the overall QoL for diabetes patients.

The present study has several limitations. It was conducted at a single, though prominent, healthcare center in Sri Lanka, which may limit the ability to generalize our findings to other populations across the country.

## Conclusions

Nearly half of the DM patients in this study experienced poor quality of life (QoL). To enhance QoL for DM patients, it is important to focus on improving their socioeconomic conditions, promoting healthier lifestyle choices, increasing awareness about diabetes prevention and management, and providing assistance with medical costs. The findings of this study reveal various factors that influence the quality of life in diabetes patients, emphasizing the need for tailored interventions that address demographic factors, lifestyle habits, complications, and comorbidities to improve health and well-being.

## Supplementary Information

Below is the link to the electronic supplementary material.

Supplementary Material 1

## Data Availability

The datasets used and/or analysed during the current study are available from the corresponding author on reasonable request.

## Abbreviations

HRQoL: Health-related Quality of life
DM: Diabetes Mellitus
LMIC: Low and middle-income countries
IDF: International Diabetes Federation
WHO: World Health Organization

## References

[1] Heald AH, Stedman M, Davies M, Livingston M, Alshames R, Lunt M, Rayman G, Gadsby R. Estimating life years lost to diabetes: outcomes from analysis of National Diabetes Audit and Office of National Statistics data. Cardiovasc Endocrinol Metab. 2020;9(4):183–5. 33225235 10.1097/XCE.0000000000000210 PMC7673790

[2] Sun H, Saeedi P, Karuranga S, Pinkepank M, Ogurtsova K, Duncan BB, Stein C, Basit A, Chan JCN, Mbanya JC, et al. IDF Diabetes Atlas: Global, regional and country-level diabetes prevalence estimates for 2021 and projections for 2045. Diabetes Res Clin Pract. 2022;183:109119. 34879977 10.1016/j.diabres.2021.109119 PMC11057359

[3] International Diabetes Federation. IDF Diabetes Atlas. 9th ed. Brussels, Belgium: International Diabetes Federation; 2019.

[4] Rannan-Eliya RP, Wijemunige N, Perera P, Kapuge Y, Gunawardana N, Sigera C, Jayatissa R, Herath HMM, Gamage A, Weerawardena N, et al. Prevalence of diabetes and pre-diabetes in Sri Lanka: a new global hotspot-estimates from the Sri Lanka Health and Ageing Survey 2018/2019. BMJ Open Diabetes Res Care. 2023;11(1):e003160. 36796852 10.1136/bmjdrc-2022-003160 PMC9936281

[5] Hippisley-Cox J, Coupland C. Diabetes treatments and risk of amputation, blindness, severe kidney failure, hyperglycaemia, and hypoglycaemia: open cohort study in primary care. BMJ. 2016;352:i1450. 27029547 10.1136/bmj.i1450 PMC4816603

[6] Kalra S, Jena BN, Yeravdekar R. Emotional and Psychological Needs of People with Diabetes. Indian J Endocrinol Metab. 2018;22(5):696–704. 30294583 10.4103/ijem.IJEM_579_17 PMC6166557

[7] Santhalingam S, Sivagurunathan S, Prathapan S, Kanagasabai S, Kamalarupan L. The effect of socioeconomic factors on the quality of life of the elderly in the Jaffna district of Sri Lanka. PLOS Glob Public Health. 2022;2(8):e0000916. 36962835 10.1371/journal.pgph.0000916 PMC10021859

[8] Ma X. McGhee SMA cross-sectional study on socioeconomic status and health-related quality of life among elderly Chinese. BMJ Open. 2013;3:e002418. 23377996 10.1136/bmjopen-2012-002418 PMC3586150

[9] Llorens-Ortega R, Bertran-Noguer C, Juvinyà-Canals D, Garre-Olmo J, Bosch-Farré C. Influence of social determinants of health in the evolution of the quality of life of older adults in Europe: A comparative analysis between men and women. Humanit Soc Sci Commun. 2024;11:401.

[10] Wong CK, Lo YY, Wong WH, Fung CS. The associations of body mass index with physical and mental aspects of health-related quality of life in Chinese patients with type 2 diabetes mellitus: results from a cross-sectional survey. Health Qual Life Outcomes. 2013;11(1):1–9. 23964785 10.1186/1477-7525-11-142 PMC3765933

[11] Qaseem A, Vijan S, Snow V, Cross JT, Weiss KB, Owens DK. Glycemic Control and Type 2 Diabetes Mellitus: the Optimal Hemoglobin A1c Targets. A Guidance Statement from the American College of Physicians. Ann Intern Med. 2007;147(6):417–22. 17876024 10.7326/0003-4819-147-6-200709180-00012

[12] Zurita-Cruz JN, Manuel-Apolinar L, Arellano-Flores ML, Gutiérrez-González A, Nájera-Ahumada AG, Cisneros-González N. Health and quality of life outcomes impairment of quality of life in type 2 diabetes mellitus: a cross-sectional study. Health Qual Life Outcomes. 2018;16(1):94. 29764429 10.1186/s12955-018-0906-y PMC5952418

[13] Quah JH, Luo N, Ng WY, How CH, Tay EG. Health-related quality of life is associated with diabetic complications, but not with short-term diabetic control in primary care. Ann Acad Med Singap. 2011;40(6):276–86. 21779616

[14] Adriaanse MC, Drewes HW, van der Heide I, Struijs JN, Baan CA. The impact of comorbid chronic conditions on quality of life in type 2 diabetes patients. Qual Life Res. 2016;25(1):175–82. 26267523 10.1007/s11136-015-1061-0 PMC4706581

[15] Alaryni A. Assessment and Factors Contributing to the Quality of Life in Diabetes Mellitus Patients: A Cross-Sectional Single-Center Study. Cureus. 2024;16(2):e54359. 38500939 10.7759/cureus.54359 PMC10945466

[16] Skevington SM, Lotfy M, O’Connell KA. The World Health Organization’s WHOQOL-BREF quality of life assessment: Psychometric properties and results of the international field trial. Qual Life Research: Int J Qual Life Aspects Treat Care Rehabilit. 2004;13(2):299–310. 10.1023/B:QURE.0000018486.91360.00 15085902

[17] The WHOQOL Group. Development of the World Health Organization WHOQOL-BREF Quality of Life Assessment. Psychol Med. 1998;28:551–8. 9626712 10.1017/s0033291798006667

[18] Kumarapeli V, Senevirathne R, Wijayarathna CN. Validation of WHOQOL-BREF to measure quality of life among women with polycystic ovary syndrome. J Coll Community Physicians Sri Lanka. 2006;11(2):1–9.

[19] Suzanne M. Skevington · Norman Sartorius · Marianne Amir and The WHOQOL-Group 1, Developing methods for assessing quality of life in different cultural settings: The history of the WHOQOL instruments. Soc Psychiatry Psychiatr Epidemiol. 2004;39:1–8. 15022040 10.1007/s00127-004-0700-5

[20] Kanaley JA, Colberg SR, Corcoran MH, Malin SK, Rodriguez NR, Crespo CJ, Kirwan JP, Zierath JR. Exercise/Physical Activity in Individuals with Type 2 Diabetes: A Consensus Statement from the American College of Sports Medicine. Med Sci Sports Exerc. 2022;54(2):353–68. 35029593 10.1249/MSS.0000000000002800 PMC8802999

[21] Sugandh F, Chandio M, Raveena F, Kumar L, Karishma F, Khuwaja S, Memon UA, Bai K, Kashif M, Varrassi G, Khatri M, Kumar S. Advances in the Management of Diabetes Mellitus: A Focus on Personalized Medicine. Cureus. 2023;15(8):e43697. 37724233 10.7759/cureus.43697 PMC10505357

[22] Nguyen HTT, Moir MP, Nguyen TX, Vu AP, Luong LH, Nguyen TN, Nguyen LH, Tran BX, Tran TT, Latkin CA, et al. Health-related quality of life in elderly diabetic outpatients in Vietnam. Patient Preference Adherence. 2018;12:1347–54. 30100711 10.2147/PPA.S162892 PMC6067618

[23] Wan EY, Fung CS, Choi EP, Wong CK, Chan AK, Chan KH, Lam CL. Main predictors in health-related quality of life in Chinese patients with type 2 diabetes mellitus. Qual Life Res. 2016;25(11):2957–65. 27299744 10.1007/s11136-016-1324-4

[24] Lee SH. The Growing Challenge of Diabetes Management in an Aging Society. Diabetes Metab J. 2023;47(5):630–1. 37793980 10.4093/dmj.2023.0279 PMC10555539

[25] Amarasinghe T, Kumara D, Damayanthi H, Warnasooriya W, Antonypillai C. Health related quality of life of the diabetic patients attending a tertiary care hospital in central province of Sri Lanka. In: General Sir John Kotelawala Defence University – 14th international research conference. 2021;55.

[26] Gupta J, Kapoor D, Sood V. Quality of Life and its Determinants in Patients with Diabetes Mellitus from Two Health Institutions of Sub-Himalayan Region of India. Indian J Endocrinol Metab. 2021;25:211–9. 34760676 10.4103/ijem.IJEM_246_21 PMC8547395

[27] Thapa S, Pyakurel P, Baral DD, Jha N. Health-related quality of life among people living with type 2 diabetes: A community-based cross-sectional study in rural Nepal. BMC Public Health. 2016;19:1–6. 10.1186/s12889-019-7506-6 PMC6712607 31455280

[28] Abedini M, Bijari B, Miri Z, Shakhs F, Abbasi A. The quality of life of the patients with type 2 diabetes using EQ-5D-5 L in Birjand. Health Qual Life Outcome. 2020;18(1):1–9. 10.1186/s12955-020-1277-8 PMC6990543 32000785

[29] Gebremariam GT, Biratu S, Alemayehu M, Welie AG, Beyene K, Sander B, Gebretekle GB. Health-related quality of life of patients with type 2 diabetes mellitus at a tertiary care hospital in Ethiopia. PLoS ONE. 2022;17:1–15. 10.1371/journal.pone.0264199 PMC8856533 35180266

[30] Paterick TE, Patel N, Tajik AJ, Chandrasekaran K. Improving health outcomes through patient education and partnerships with patients. Proc (Bayl Univ Med Cent). 2017;30(1):112–3. 28152110 10.1080/08998280.2017.11929552 PMC5242136

[31] Barua L, Faruque M, Chowdhury HA, Banik PC, Ali L. Health-related quality of life and its predictors among the type 2 diabetes population of Bangladesh: A nation-wide cross-sectional study. J Diabetes Invest. 2021;12:277–85. 10.1111/jdi.13331 PMC7858106 32564501

[32] Schuch FB, Vancampfort D. Physical activity, exercise, and mental disorders: it is time to move on. Trends Psychiatry Psychother. 2021;43(3):177–84. 33890431 10.47626/2237-6089-2021-0237 PMC8638711

[33] Oyewole OO, Ale AO, Ogunlana MO, Gurayah T. Burden of disability in type 2 diabetes mellitus and the moderating effects of physical activity. World J Clin Cases. 2023;11(14):3128–39. 37274052 10.12998/wjcc.v11.i14.3128 PMC10237122

[34] Afroz A, Zhang W, Wei Loh AJ, Jie Lee DX, Billah B. Macro- and micro-vascular complications and their determinants among people with type 2 diabetes in Bangladesh. Diabetes Metabolic Syndrome: Clin Res Rev. 2019;13(5):2939–46. 10.1016/j.dsx.2019.07.046 31425960

[35] Gu S, Wang X, Shi L, Sun Q, Hu X, Gu Y. Health-related quality of life of type 2 diabetes patients hospitalized for a diabetes-related complication. Qual Life Res. 2020;29(10):2695–704. 32410144 10.1007/s11136-020-02524-3

[36] Karami H, Shirvani Shiri M, Rezapour A, Sarvari Mehrabadi R, Afshari S. The association between diabetic complications and health-related quality of life in patients with type 2 diabetes: A cross-sectional study from Iran. Qual Life Res. 2021;30(7):1963–74. 33900519 10.1007/s11136-021-02792-7

[37] Sitlinger A, Zafar SY. Health-Related Quality of Life: The Impact on Morbidity and Mortality. Surg Oncol Clin N Am. 2018;27(4):675–84. 30213412 10.1016/j.soc.2018.05.008 PMC6428416

[38] Aarthy R, Mikocka-Walus A, Pradeepa R, Anjana RM, Mohan V, Aston-Mourney K. Quality of Life and Diabetes in India: A Scoping Review. Indian J Endocrinol Metab. 2021;25(5):365–80. 35300441 10.4103/ijem.ijem_336_21 PMC8923323

[39] Trikkalinou A, Papazafiropoulou AK, Melidonis A. Type 2 diabetes and quality of life. World J Diabetes. 2017;8(4):120–9. 28465788 10.4239/wjd.v8.i4.120 PMC5394731

[40] Alpers SE, Druckrey-Fiskaaen KT, Madebo T, Vold JH, Pallesen S, Skogen JC, Lunde LH, Mæland S, Fadnes LT. The association of psychological distress and economic and health worries with tobacco smoking behavior during the COVID-19 pandemic: a two-year longitudinal cohort study. BMC Public Health. 2024;24(1):375. 38317145 10.1186/s12889-024-17943-x PMC10840189

[41] Campagna D, Alamo A, Di Pino A, Russo C, Calogero AE, Purrello F, Polosa R. Smoking and diabetes: dangerous liaisons and confusing relationships. Diabetol Metab Syndr. 2019;11:85. 31666811 10.1186/s13098-019-0482-2 PMC6813988

[42] Maddatu J, Anderson-Baucum E, Evans-Molina C. Smoking and the risk of type 2 diabetes. Transl Res. 2017;184:101–7. 28336465 10.1016/j.trsl.2017.02.004 PMC5429867

[43] Mansfield KJ, Colicchio VD, Kauwe Tuitama AI, Tracy EL, Neuberger JD, Litchman ML. Care Partner Support Following a Diabetes Self-Management Education and Support Intervention. Sci Diabetes Self Manag Care. 2022;48(4):235–46. 35658746 10.1177/26350106221099872 PMC10120568

[44] Wrzus C, Hänel M, Wagner J, Neyer FJ. Social network changes and life events across the life span: a meta-analysis. Psychol Bull. 2013;139(1):53-80. 10.1037/a0028601 22642230

