# Supplementary material for: Health-related quality of life and its determinants among patients with diabetes mellitus in a selected hospital in Sri Lanka: a cross-sectional study
Source: BMC Endocr Disord. 2026 Aug 10;26:270. doi: 10.1186/s12902-026-02476-8 (PMC13573390; doi:10.1186/s12902-026-02476-8)
Supplement: Supplementary file 1 [file 12902_2026_2476_MOESM1_ESM.pdf]

a54445

WHOQOL-BREF

INTRODUCTION, ADMINISTRATION, SCORING AND GENERIC VERSION OF THE ASSESSMENT

Field Trial Version
December 1996

PROGRAMME ON MENTAL HEALTH

WORLD HEALTH ORGANIZATION

GENEVA

This manual was drafted by Alison Harper on behalf of the WHOQOL group. The WHOQOL group comprises a coordinating group, collaborating investigators in each of the field centres and a panel of consultants. Dr J. Orley directs the project. He has been assisted in this by Professor M. Power, Dr W. Kuyken, Professor N. Sartorius, Dr M. Bullinger and Dr A. Harper. The field centres involved in initial piloting of the WHOQOL were: Professor H. Herrman, Dr H. Schofield and Ms B. Murphy, University of Melbourne, Australia; Professor Z. Metelko, Professor S. Szabo and Mrs M. Pibernik-Okanovic, Institute of Diabetes, Endocrinology and Metabolic Diseases and Department of Psychology, Faculty of Philosophy, University of Zagreb, Croatia; Dr N. Quemada and Dr A. Caria, INSERM, Paris, France; Dr S. Rajkumar and Mrs Shuba Kumar, Madras Medical College, India; Dr S. Saxena and Dr K. Chandiramani, All India Institute of Medical Sciences, New Delhi, India; Dr M. Amir and Dr D. Bar-On, Ben-Gurion University of the Negev, Beer-Sheeva, Israel; Dr Miyako Tazaki, Department of Science, Science University of Tokyo, Japan and Dr Ariko Noji, Department of Community Health Nursing, St Luke's College of Nursing, Japan; Dr G. van Heck and Mrs J. De Vries, Tilburg University, The Netherlands; Professor J. Arroyo Sucre and Professor L. Picard-Ami, University of Panama, Panama; Professor M. Kabanov, Dr A. Lomachenkov and Dr G. Burkovsky, Bekhterev Psychoneurological Research Institute, St. Petersburg, Russia; Dr R. Lucas Carrasco, University of Barcelona, Spain; Dr Yooth Bodharamik and Mr Kitikorn Meesapya, Institute of Mental Health, Bangkok, Thailand; Dr S. Skevington, University of Bath, United Kingdom; Professor D. Patrick, Ms M. Martinand, Ms D. Wild, University of Washington, Seattle, USA and; Professor W. Acuda and Dr J. Mutambirwa, University of Zimbabwe, Harare, Zimbabwe.

New centres using the field version of the WHOQOL-100 are: Dr S. Bonicato, FUNDONAR, Fundacion Oncologica Argentina, Argentina; Dr A.E. Molzahn, University of Victoria, Canada; Dr G. Yongping, St Vincent's Hospital, Victoria, Australia; Dr G. Page, University of Quebec at Rimouski, Canada; Professor J. Fang, Sun Yat-Sen University of Medical Sciences, People's Republic of China; Dr M. Fleck, University of the State of Rio Grande do Sul, Brazil; Professor M.C. Angermeyer, Dr R. Kilian, Universitätsklinikum Klinik und Poliklinik für Psychiatrie, Leipzig, Germany; Mr L. Kwok-fai, Queen Elizabeth Hospital, Kowloon, Hong Kong; Dr B.R. Hanestad, University of Bergen, Norway; Dr M.H. Mubbashar, Rawalpindi General Hospital, Pakistan; Dr J. Harangozo, Semelweis University of Medicine, Budapest & Dr L. Kullman, National Institute of Mental Rehabilitation, Budapest, Hungary; Professor I. Wiklund, Health Economics & Quality of Life, Astra Hässle AB, Sweden; Dr C. Fidaner, Dr Behçet Uz Paediatric Hospital, Balçova/Izmir, Turkey; Dr G. de Girolamo, Servizio Salute Mentale USL 27, Italy; Professor P. Bech, Frederiksborg General Hospital, Denmark; Dr R.S. Pippalla, Howard University, College of Pharmacy and Pharmaceutical Sciences, Washington, DC, USA and Dr H. Che Ismail, School of Medical Sciences, Kelantan, Malaysia.

Further information can be obtained from:
Dr John Orley
Programme on Mental Health
World Health Organization
CH-1211 Geneva 27, Switzerland

This document is not issued to the general public, and all rights are reserved by the World Health Organization (WHO). This document may not be reviewed, abstracted, quoted, reproduced, translated, referred to in bibliographical matter or cited, in part or in whole, without the prior written permission of WHO. No part of this document may be stored in a retrieval system or transmitted in any form by any means - electronic, mechanical or other -without the prior written permission of WHO. The WHOQOL Group, Programme on Mental Health, WHO, CH-1211 Geneva 27, Switzerland.

WHOQOL-BREF
Introduction, Administration, Scoring and Generic Version of the Assessment

Introduction

The WHOQOL-100 quality of life assessment was developed by the WHOQOL Group with fifteen international field centres, simultaneously, in an attempt to develop a quality of life assessment that would be applicable cross-culturally. The development of the WHOQOL-100, has been detailed elsewhere (i.e. Orley & Kuyken, 1994; Szabo, 1996; WHOQOL Group 1994a, 1994b, 1995). This document gives a conceptual background to the WHOQOL definition of quality of life and describes the development of the WHOQOL-BREF, an abbreviated version of the WHOQOL-100. It also includes a generic English language version of the WHOQOL-BREF, instructions for administering and scoring, and proposed uses for this short form of the WHOQOL.

Rationale for the development of the WHOQOL-100

WHO's initiative to develop a quality of life assessment arose for a number of reasons. In recent years there has been a broadening in focus in the measurement of health, beyond traditional health indicators such as mortality and morbidity (e.g. World Bank, 1993; WHO, 1991), to include measures of the impact of disease and impairment on daily activities and behaviour (e.g. Sickness Impact Profile; Bergner, Bobbitt, Carter et al, 1981), perceived health measures (e.g. Nottingham Health Profile; Hunt, McKenna and McEwan, 1989) and disability / functional status measures (e.g. the MOS SF-36, Ware et al, 1993). These measures, whilst beginning to provide a measure of the impact of disease, do not assess quality of life *per se*, which has been aptly described as "the missing measurement in health" (Fallowfield, 1990). Second, most measures of health status have been developed in North America and the UK, and the translation of these measures for use in other settings is time-consuming, and unsatisfactory for a number of reasons (Sartorius and Kuyken, 1994; Kuyken, Orley, Hudelson and Sartorius, 1994). Third, the increasingly mechanistic model of medicine, concerned only with the eradication of disease and symptoms, reinforces the need for the introduction of a humanistic element into health care. By calling for quality of life assessments in health care, attention is focused on this aspect of health, and resulting interventions will pay increased attention to this aspect of patients' well-being. WHO's initiative to develop a quality of life assessment arises from a need for a genuinely international measure of quality of life and a commitment to the continued promotion of an holistic approach to health and health care.

Steps in the development of the WHOQOL-100

The WHOQOL-100 development process consisted of several stages. These are explained in brief within this document. For a detailed description, the reader is referred to the WHOQOL Group (1994a, 1994b, in preparation). In the first stage, concept clarification involved establishing an agreed upon definition of quality of life and an approach to international quality of life assessment.

Quality of life is defined as individuals' perceptions of their position in life in the context of the culture and value systems in which they live and in relation to their goals, expectations, standards and concerns.

This definition reflects the view that quality of life refers to a subjective evaluation which is embedded in a cultural, social and environmental context. Because this definition of quality of life focuses upon respondents' "perceived" quality of life, it is not expected to provide a means of measuring in any detailed fashion symptoms, diseases or conditions, but rather the effects of disease and health interventions on quality of life. As such, quality of life cannot be equated simply with the terms "health status", "life style", "life satisfaction", "mental state" or "well-being". The recognition of the multi-dimensional nature of quality of life is reflected in the WHOQOL-100 structure.

In the second stage of development, exploration of the quality of life construct within 15 culturally diverse field centres was carried out to establish a list of areas/facets that participating centres considered relevant to the assessment of quality of life. This involved a series in meetings of focus groups which included health professionals, patients and well subjects. A maximum of six specific items for exploring each proposed facet were generated by each centre's focus group. To enable the collaboration to be genuinely international the 15 field centres were selected world-wide to provide differences in level of industrialisation, available health services, and other markers relevant to the measurement of quality of life (e.g. role of the family, perception of time, perception of self, dominant religion).

In the third stage of development, questions from each centre were assembled into a global pool. After clustering semantically equivalent questions, 236 items covering 29 facets were included in a final assessment. Pilot work involved administration of this standardised assessment to at least 300 respondents within each centre.

Following field testing in these 15 centres, 100 items were selected for inclusion in the WHOQOL-100 Field Trial Version. These included four items for each of 24 facets of quality of life, and four items relating to the 'overall quality of life and general health' facet (see Table 1). The method by which these 100 items were selected is fully documented elsewhere (The WHOQOL Group, in preparation). The WHOQOL-100 Field Trial Version is currently being tested in new centres world-wide (these centres are outlined on page 6 of this document). The initial conceptual framework for the WHOQOL-100 proposed that the 24 facets relating to quality of life should be grouped into 6 domains. Recent analysis of available data, using structural equation modelling, has shown a four domain solution to be more appropriate. For a more detailed explanation of this, the reader is referred to The WHOQOL Group (in preparation). The WHOQOL-BREF is therefore based on a four domain structure (see Table 1).

Table 1 - WHOQOL-BREF domains

Domain	Facets incorporated within domains
1. Physical health	Activities of daily living<br>Dependence on medicinal substances and medical aids<br>Energy and fatigue<br>Mobility<br>Pain and discomfort<br>Sleep and rest<br>Work Capacity
2. Psychological	Bodily image and appearance<br>Negative feelings<br>Positive feelings<br>Self-esteem<br>Spirituality / Religion / Personal beliefs<br>Thinking, learning, memory and concentration
3. Social relationships	Personal relationships<br>Social support<br>Sexual activity
4. Environment	Financial resources<br>Freedom, physical safety and security<br>Health and social care: accessibility and quality<br>Home environment<br>Opportunities for acquiring new information and skills<br>Participation in and opportunities for recreation / leisure activities<br>Physical environment (pollution / noise / traffic / climate)<br>Transport

Development of the WHOQOL-BREF

The WHOQOL-100 allows detailed assessment of each individual facet relating to quality of life. In certain instances however, the WHOQOL-100 may be too lengthy for practical use. The WHOQOL-BREF Field Trial Version has therefore been developed to provide a short form quality of life assessment that looks at Domain level profiles, using data from the pilot WHOQOL assessment and all available data from the Field Trial Version of the WHOQOL-100. Twenty field centres situated within eighteen countries have included data for these purposes (see Table 2). The WHOQOL-BREF contains a total of 26 questions. To provide a broad and comprehensive assessment, one item from each of the 24 facets contained in the WHOQOL-100 has been included. In addition, two items from the Overall quality of Life and General Health facet have been included.

Table 2 - Centres included in development of the WHOQOL-BREF

Centres in the pilot version of the WHOQOL	Centres in the field trial of the WHOQOL-100
Bangkok, Thailand	Bangkok, Thailand
Beer Sheva, Israel	Beer Sheva, Israel
Madras, India	Madras, India
Melbourne, Australia	Melbourne, Australia
New Delhi, India	New Delhi, India
Panama City, Panama	Panama City, Panama
Seattle, USA	Seattle, USA
Tilburg, The Netherlands	Tilburg, The Netherlands
Zagreb, Croatia	Zagreb, Croatia
Tokyo, Japan	Tokyo, Japan
Harare, Zimbabwe	Harare, Zimbabwe
Barcelona, Spain	Barcelona, Spain
Bath, UK	Bath, UK
St Petersburg, Russia	Kowloon, Hong Kong
Paris, France	Leipzig, Germany
	Mannheim, Germany
	La Plata, Argentina
	Port Alegre, Brazil

The WHOQOL-BREF is available in 19 different languages. The appropriate language version, and permission for using it, can be obtained from The WHOQOL Group, Programme on Mental Health, World Health Organisation, CH-1211 Geneva 27, Switzerland. Under no circumstances should the WHOQOL-BREF be used without consultation with The WHOQOL Group. A methodology has been developed for new centres wishing to develop a further language version of the WHOQOL-100 or the WHOQOL-BREF. This can be obtained from The WHOQOL Group, Programme on Mental Health, World Health Organisation, CH-1211, Geneva 27, Switzerland.

Questions should appear in the order in which they appear in the example WHOQOL-BREF provided within this document, with instructions and headers unchanged. Questions are grouped by response format. The equivalent numbering of questions between the WHOQOL-BREF and the WHOQOL-100 is given in the example version of the WHOQOL-BREF to enable easy comparison between responses to items on the two versions. The WHOQOL-100 field test permitted centres to include national items or facets that were thought to be important in assessing quality of life. Where centres wish to include additional national items or modules to the WHOQOL-BREF, these should be included on a separate sheet of paper and not scattered amongst the existing 26 items. There are three reasons for this:

1) To control for item order effects which could occur and change item meaning.
2) The WHOQOL-BREF represents an agreed upon core set of international items.
3) The WHOQOL-BREF is likely to be used where quality of life is amongst one of several parameters being assessed. Therefore additional national information can be obtained by including additional modules and measures

Administration of the WHOQOL-BREF

For any new centre not previously involved in either the development or field testing of the WHOQOL-100, the procedure being followed to field test the WHOQOL-BREF should be identical to that used to field test the WHOQOL-100. The instrument should be piloted on at least 300 people. This figure is based on the required numbers of respondents needed for analysis of pilot data. The sample of respondents to whom the assessment should be administered ought to be adults, with 'adult' being culturally defined. While stratified samples are not essential, a sampling quota should apply with regard to:

- Age (50% = <45 years, 50% = 45+ years)
- Sex (50% = male, 50% = female)
- Health status (250 persons with disease or impairment; 50 well persons)

With respect to persons with disease or impairment, this group should contain a cross-section of people with varied levels of quality of life. One way of attempting this would be to include some people with quite severe and disabling chronic diseases, some people in contact with health facilities for more transient conditions, possibly some attending a family practitioner, and others who are in contact with the health service for reasons that are not likely to impinge upon their quality of life to any great extent. By sampling patients from a cross-section of primary care settings, hospitals and community care settings this could most likely be achieved.

The WHOQOL-BREF should be self-administered if respondents have sufficient ability: otherwise, interviewer-assisted or interview-administered forms should be used. Standardised instructions, given on the second page of the WHOQOL-BREF example assessment, should be read out to respondents in instances where the assessment is interviewer-administered.

For centres who have already participated in the development and field testing of the WHOQOL-100, the above option of testing the WHOQOL-BREF is preferred, but not imperative where specific studies of patient groups are planned.

Frame of reference and time frame

A time frame of two weeks is indicated in the assessment. It is recognised that different time frames may be necessary for particular uses of the instrument in subsequent stages of work. For example, in the assessment of quality of life in chronic conditions, such as arthritis, a longer time frame such as four weeks may be preferable. Furthermore, the perception of time is different within different cultural settings and therefore changing the time scale may be appropriate.

Proposed uses of the WHOQOL-100 and the WHOQOL-BREF

It is anticipated that the WHOQOL assessments will be used in broad-ranging ways. They will be of considerable use in clinical trials, in establishing baseline scores in a range of areas, and looking at changes in quality of life over the course of interventions. It is expected that the WHOQOL assessments will also be of value where disease prognosis is likely to involve only partial recovery or remission, and in which treatment may be more palliative than curative.

For epidemiological research, the WHOQOL assessments will allow detailed quality of life data to be gathered on a particular population, facilitating the understanding of diseases, and the development of treatment methods. The international epidemiological studies that would be enabled by instruments such as the WHOQOL-100 and the WHOQOL-BREF will make it possible to carry out multi-centre quality of life research, and to compare results obtained in different centres. Such research has important benefits, permitting questions to be addressed which would not be possible in single site studies (Sartorius and Helmchen, 1981). For example, a comparative study in two or more countries on the relationship between health care delivery and quality of life requires an assessment yielding cross-culturally comparable scores. Sometimes accumulation of cases in quality of life studies, particularly when studying rare disorders, is helped by gathering data in several settings. Multi-centre collaborative studies can also provide simultaneous multiple replications of a finding, adding considerably to the confidence with which findings can be accepted.

In clinical practice the WHOQOL assessments will assist clinicians in making judgements about the areas in which a patient is most affected by disease, and in making treatment decisions. In some developing countries, where resources for health care may be limited, treatments aimed at improving quality of life through palliation, for example, can be both effective and inexpensive (Olweny, 1992). Together with other measures, the WHOQOL-BREF will enable health professionals to assess changes in quality of life over the course of treatment.

It is anticipated that in the future the WHOQOL-100 and the WHOQOL-BREF will prove useful in health policy research and will make up an important aspect of the routine auditing of health and social services. Because the instrument was developed cross-culturally, health care providers, administrators and legislators in countries where no validated quality of life measures currently exist can be confident that data yielded by work involving the WHOQOL assessments will be genuinely sensitive to their setting.

Scoring the WHOQOL-BREF

The WHOQOL-BREF (Field Trial Version) produces a quality of life profile. It is possible to derive four domain scores. There are also two items that are examined separately: question 1 asks about an individual's overall perception of quality of life and question 2 asks about an individual's overall perception of their health. The four domain scores denote an individual's perception of quality of life in each particular domain. Domain scores are scaled in a positive direction (i.e. higher scores denote higher quality of life). The mean score of items within each domain is used to calculate the domain score. Mean scores are then multiplied by 4 in order to make domain scores comparable with the scores used in the WHOQOL-100. Explicit instructions for checking and cleaning data, and for computing domain scores, are given in Table 3. A method for the manual calculation of individual scores is given on page 1 of the WHOQOL-BREF assessment form. The method for converting raw scores to transformed scores when using this method is given in Table 4, on page 11 of these instructions. The first transformation method converts scores to range between 4-20, comparable with the WHOQOL-100. The second transformation method converts domain scores to a 0-100 scale.

Where more than 20% of data is missing from a assessment, the assessment should be discarded (see Step 4 in Table 3). Where an item is missing, the mean of other items in the domain is substituted. Where more than two items are missing from the domain, the domain score should not be calculated (with the exception of domain 3, where the domain should only be calculated if ≤ 1 item is missing).

Any national items should be scored separately from the core 26 item of the BREF. During the analysis the performance of any national items will be examined for possible use in alter national studies. At this stage of field testing national and core items must not be mixed in administration or scoring of the BREF.

An SPSS syntax file that automatically checks, recodes data and computes domain scores may be obtained from Professor Mick Power, Department of Psychiatry, Royal Edinburgh Hospital, Morningside Park, Edinburgh, EH10 5HF (email: mj@srv2.med.ed.ac.uk; fax: + 131 447 6860)

Table 3 - Steps for checking and cleaning data and computing domain scores

	Steps	SPSS syntax for carrying out data checking, cleaning and computing total scores
1.	Check all 26 items from assessment have a range of 1-5	RECODE Q1 Q2 Q3 Q4 Q5 Q6 Q7 Q8 Q9 Q10 Q11 Q12 Q13 Q14 Q15 Q16 Q17 Q18 Q19 Q20 Q21 Q22 Q23 Q24 Q25 Q26 (1=1) (2=2) (3=3) (4=4) (5=5) (ELSE=SYSMIS).<br>(This recodes all data outwith the range 1-5 to system missing).
2.	Reverse 3 negatively phrased items	RECODE Q3 Q4 Q26 (1=5) (2=4) (3=3) (4=2) (5=1).<br>(This transforms negatively framed questions to positively framed questions)
3.	Compute domain scores	COMPUTE DOM1=MEAN.6(Q3,Q4,Q10,Q15,Q16,Q17,Q18)*4.<br>COMPUTE DOM2=MEAN.5(Q5,Q6,Q7,Q11,Q19,Q26)*4.<br>COMPUTE DOM3=MEAN.2(Q20,Q21,Q22)*4.<br>COMPUTE DOM4=MEAN.6(Q8,Q9,Q12,Q13,Q14,Q23,Q24,Q25)*4.<br>(These equations calculate the domain scores. All scores are multiplied by 4 so as to be directly comparable with scores derived from the WHOQOL-100. The '.6' in 'mean.6' specifies that 6 items must be endorsed for the domain score to be calculated).
4.	Delete cases with >20% missing data	COUNT TOTAL=Q1 TO Q26 (1 THRU 5).<br>(This command creates a new column 'total'. 'Total' contains a count of the WHOQOL-100 items with the values 1-5 that have been endorsed by each subject. The 'Q1 TO Q26' means that consecutive columns from 'Q1', the first item, to 'Q26', the last item, are included in the count. It therefore assumes that data is entered in the order given in the assessment).<br>FILTER OFF.<br>USE ALL.<br>SELECT IF (TOTAL>=21).<br>EXECUTE.<br>(This second command selects only those cases where 'total', the total number of items completed, is greater or equal to 80%. It deletes the remaining cases from the data set).
5.	Check domain scores	DESCRIPTIVES<br>VARIABLES=DOM1 DOM2 DOM3 DOM4<br>/STATISTICS=MEAN STDDEV MIN MAX.<br>(Running descriptives should display values of all domain scores within the range 4-20).
6.	Save data set	Save data set with a new file name so that the original remains intact.

Table 4 - Method for converting raw scores to transformed scores

DOMAIN 1		
Raw score	Trasnformed scores	
	4-20	0-100
7	4	0
8	5	6
9	5	6
10	6	13
11	6	13
12	7	19
13	7	19
14	8	25
15	9	31
16	9	31
17	10	38
18	10	38
19	11	44
20	11	44
21	12	50
22	13	56
23	13	56
24	14	63
25	14	63
26	15	69
27	15	69
28	16	75
29	17	81
30	17	81
31	18	88
32	18	88
33	19	94
34	19	94
35	20	100

DOMAIN 2		
Raw score	Trasnformed scores	
	4-20	0-100
6	4	0
7	5	6
8	5	6
9	6	13
10	7	19
11	7	19
12	8	25
13	9	31
14	9	31
15	10	38
16	11	44
17	11	44
18	12	50
19	13	56
20	13	56
21	14	63
22	15	69
23	15	69
24	16	75
25	17	81
26	17	81
27	18	88
28	19	94
29	19	94
30	20	100

DOMAIN 3		
Raw score	Transformed scores	
	4-20	0-100
3	4	0
4	5	6
5	7	19
6	8	25
7	9	31
8	11	44
9	12	50
10	13	56
11	15	69
12	16	75
13	17	81
14	19	94
15	20	100

DOMAIN 4		
Raw score	Transformed scores	
	4-20	0-100
8	4	0
9	5	6
10	5	6
11	6	13
12	6	13
13	7	19
14	7	19
15	8	25
16	8	25
17	9	31
18	9	31
19	10	38
20	10	38
21	11	44
22	11	44
23	12	50
24	12	50
25	13	56
26	13	56
27	14	63
28	14	63
29	15	69
30	15	69
31	16	75
32	16	75
33	17	81
34	17	81
35	18	88
36	18	88
37	19	94
38	19	94
39	20	100
40	20	100

References

Bergner, M., Bobbitt, R.A., Carter, W.B. *et al.* (1981). The Sickness Impact Profile: Development and final revision of a health status measure. *Medical Care*, **19**, 787-805.

Fallowfield, L. (1990). *The Quality of Life: The Missing Measurement in Health Care.* Souvenir Press.

Hunt, S.M., McKenna, S.P. and McEwan, J. (1989). *The Nottingham Health Profile. Users Manual.* Revised edition.

Kuyken, W., Orley, J., Hudelson, P. and Sartorius, N. (1994). Quality of life assessment across cultures. *International Journal of Mental Health*, **23** (2), 5-27.

Olweny, C. L. M. (1992). Quality of life in developing countries. *Journal of Palliative Care*, 8, 25-30.

Sartorius, N. and Helmchen, H. (1981). Aims and implementation of multi-centre studies. *Modern Problems of Pharmacopsychiatry*, **16**, 1-8.

Sartorius, N. and Kuyken, W. (1994). Translation of health status instruments. In J. Orley and W. Kuyken (Eds). *Quality of Life Assessment: International Perspectives.* Heidelberg: Springer Verlag.

Szabo, S. (1996). The World Health Organisation Quality of Life (WHOQOL) Assessment Instrument. In *Quality of Life and Pharmaeconomics in Clinical Trials* (2nd edition, Edited by Spilker B.). Lippincott-Raven Publishers, Philadelphia, New York.

The WHOQOL Group. The World Health Organization Quality of Life assessment (WHOQOL): position paper from the World Health Organization. *Soc. Sci. Med.*, **41**, 1403, 1995.

Ware, J. E., Snow, K., K., Kosinski, M. and Gandek, B. (1993). *SF-36 Health Survey: Manual and Interpretation Guide.* New England Medical Center, MA, USA.

World Bank. (1993). *World Development Report: Investing in Health.* New York: Oxford University Press.

World Health Organization. (1991). *World Health Statistics Annual.* Geneva: WHO.

The WHOQOL Group. (1994a). Development of the WHOQOL: Rationale and current status. *International Journal of Mental Health*, **23** (3), 24-56.

The WHOQOL Group. (1994b). The development of the World Health Organization quality of life assessment instrument (the WHOQOL). In J. Orley and W. Kuyken (Eds) *Quality of Life Assessment: International Perspectives.* Heidelberg: Springer Verlag.

The WHOQOL Group. (In preparation). The World Health Organisation Quality of Life Assessment (WHOQOL): Development and General Psychometric Properties.

WHOQOL-BREF

Field Trial Version
December 1996

PROGRAMME ON MENTAL HEALTH
WORLD HEALTH ORGANIZATION
GENEVA

For office use only

	Equations for computing domain scores	Raw score	Transformed scores*	
			4-20	0-100
**Domain 1**	(6-Q3) + (6-Q4) + Q10 + Q15 + Q16 + Q17 + Q18<br>☐ + ☐ + ☐ + ☐ + ☐ + ☐ + ☐	=		
**Domain 2**	Q5 + Q6 + Q7 + Q11 + Q19 + (6-Q26)<br>☐ + ☐ + ☐ + ☐ + ☐ + ☐	=		
**Domain 3**	Q20 + Q21 + Q22<br>☐ + ☐ + ☐	=		
Domain 4	Q8 + Q9 + Q12 + Q13 + Q14 + Q23 + Q24 + Q25<br>☐ + ☐ + ☐ + ☐ + ☐ + ☐ + ☐ + ☐	=		

* Please see Table 4 on page 10 of the manual, for converting raw scores to transformed scores.

ABOUT YOU

I.D. number

Before you begin we would like to ask you to answer a few general questions about yourself: by circling the correct answer or by filling in the space provided.

What is your **gender?** Male Female

What is you **date of birth?** ________ / ________ / ________
Day / Month / Year

What is the highest **education** you received?
None at all
Primary school
Secondary school
Tertiary

What is your **marital status?**
Single
Married
Living as married
Separated
Divorced
Widowed

Are you currently **ill?** Yes No

If something is wrong with your health what do you think it is?__________________________illness/ problem

Instructions

This assessment asks how you feel about your quality of life, health, or other areas of your life. **Please answer all the questions.** If you are unsure about which response to give to a question, **please choose the one** that appears most appropriate. This can often be your first response.

Please keep in mind your standards, hopes, pleasures and concerns. We ask that you think about your life **in the last two weeks.** For example, thinking about the last two weeks, a question might ask:

		Not at all	Not much	Moderately	A great deal	Completely
	Do you get the kind of support from others that you need?	1	2	3	4	5

You should circle the number that best fits how much support you got from others over the last two weeks. So you would circle the number 4 if you got a great deal of support from others as follows.

		Not at all	Not much	Moderately	A great deal	Completely
	Do you get the kind of support from others that you need?	1	2	3	(4)	5

You would circle number 1 if you did not get any of the support that you needed from others in the last two weeks.

Please read each question, assess your feelings, and circle the number on the scale for each question that gives the best answer for you.

		Very poor	Poor	Neither poor nor good	Good	Very good
1(G1)	How would you rate your quality of life?	1	2	3	4	5

		Very dissatisfied	Dissatisfied	Neither satisfied nor dissatisfied	Satisfied	Very satisfied
2 (G4)	How satisfied are you with your health?	1	2	3	4	5

The following questions ask about **how much** you have experienced certain things in the last two weeks.

		Not at all	A little	A moderate amount	Very much	An extreme amount
3 (F1.4)	To what extent do you feel that physical pain prevents you from doing what you need to do?	1	2	3	4	5
4(F11.3)	How much do you need any medical treatment to function in your daily life?	1	2	3	4	5
5(F4.1)	How much do you enjoy life?	1	2	3	4	5
6(F24.2)	To what extent do you feel your life to be meaningful?	1	2	3	4	5

		Not at all	A little	A moderate amount	Very much	Extremely
7(F5.3)	How well are you able to concentrate?	1	2	3	4	5
8 (F16.1)	How safe do you feel in your daily life?	1	2	3	4	5
9 (F22.1)	How healthy is your physical environment?	1	2	3	4	5

The following questions ask about **how completely** you experience or were able to do certain things in the last two weeks.

		Not at all	A little	Moderately	Mostly	Completely
10 (F2.1)	Do you have enough energy for everyday life?	1	2	3	4	5
11 (F7.1)	Are you able to accept your bodily appearance?	1	2	3	4	5
12 (F18.1)	Have you enough money to meet your needs?	1	2	3	4	5
13 (F20.1)	How available to you is the information that you need in your day-to-day life?	1	2	3	4	5
14 (F21.1)	To what extent do you have the opportunity for leisure activities?	1	2	3	4	5

		Very poor	Poor	Neither poor nor good	Good	Very good
15 (F9.1)	How well are you able to get around?	1	2	3	4	5

The following questions ask you to say how **good or satisfied** you have felt about various aspects of your life over the last two weeks.

		Very dissatisfied	Dissatisfied	Neither satisfied nor dissatisfied	Satisfied	Very satisfied
16 (F3.3)	How satisfied are you with your sleep?	1	2	3	4	5
17 (F10.3)	How satisfied are you with your ability to perform your daily living activities?	1	2	3	4	5
18(F12.4)	How satisfied are you with your capacity for work?	1	2	3	4	5
19 (F6.3)	How satisfied are you with yourself?	1	2	3	4	5
20(F13.3)	How satisfied are you with your personal relationships?	1	2	3	4	5
21(F15.3)	How satisfied are you with your sex life?	1	2	3	4	5
22(F14.4)	How satisfied are you with the support you get from your friends?	1	2	3	4	5
23(F17.3)	How satisfied are you with the conditions of your living place?	1	2	3	4	5
24(F19.3)	How satisfied are you with your access to health services?	1	2	3	4	5
25(F23.3)	How satisfied are you with your transport?	1	2	3	4	5

The following question refers to **how often** you have felt or experienced certain things in the last two weeks.

		Never	Seldom	Quite often	Very often	Always
26 (F8.1)	How often do you have negative feelings such as blue mood, despair, anxiety, depression?	1	2	3	4	5

Did someone help you to fill out this form?..

How long did it take to fill this form out?..

Do you have any comments about the assessment?

....

....

THANK YOU FOR YOUR HELP
